# Effect of *Bifidobacterium breve* M-16V Supplementation on Fecal Bifidobacteria in Preterm Neonates - A Randomised Double Blind Placebo Controlled Trial

**DOI:** 10.1371/journal.pone.0089511

**Published:** 2014-03-03

**Authors:** Sanjay Patole, Anthony D. Keil, Annie Chang, Elizabeth Nathan, Dorota Doherty, Karen Simmer, Meera Esvaran, Patricia Conway

**Affiliations:** 1 Department of Neonatal Paediatrics, KEM Hospital for Women, Perth, Australia; 2 PathWest Laboratory Medicine WA, KEM Hospital for Women, Perth, Australia; 3 Women and Infants Research Foundation, KEM Hospital for Women, Perth, Australia; 4 School of Women's and Infants' Health, University of New South Wales, Sydney, Australia; 5 Centre for Neonatal Research and Education, University of Western Australia, Perth, Australia; 6 University of New South Wales, Sydney, Australia; University of Ottawa, Canada

## Abstract

**Background:**

Probiotic supplementation significantly reduces the risk of necrotising enterocolitis (NEC) and all cause mortality in preterm neonates. Independent quality assessment is important before introducing routine probiotic supplementation in this cohort.

**Aim:**

To assess product quality, and confirm that *Bifidobacterium breve* (*B. breve*) M-16V supplementation will increase fecal *B. breve* counts without adverse effects.

**Methods and Participants:**

Strain identity (16S rRNA gene sequencing), viability over 2 year shelf-life were confirmed, and microbial contamination of the product was ruled out. In a controlled trial preterm neonates (Gestation <33 weeks) ready to commence or on feeds for <12 hours were randomly allocated to either *B. breve* M-16V (3×10^9^ cfu/day) or placebo (dextrin) supplementation until the corrected age 37 weeks. Stool samples were collected before (S1) and after 3 weeks of supplementation (S2) for studying fecal *B. breve* levels using quantitative PCR (Primary outcome). Secondary outcomes included total fecal bifidobacteria and NEC≥Stage II. Categorical and continuous outcomes were analysed using Chi-square and Mann-Whitney tests, and McNemar and Wilcoxon signed-rank tests for paired comparisons.

**Results:**

A total of 159 neonates (Probiotic: 79, Placebo: 80) were enrolled. Maternal and neonatal demographic characteristics were comparable between the groups. The proportion of neonates with detectable *B. breve* increased significantly post intervention: Placebo: [S1:2/66 (3%), S2: 25/66 (38%), p<0.001] Probiotic: [S1: 29/74 (40%), S2: 67/74 (91%), p<0.001].

Median S1 *B. breve* counts in both groups were below detection (<4.7 log cells.g^−1^), increasing significantly in S2 for the probiotic group (log 8.6) while remaining <4.7 log in the control group (p<0.001). There were no adverse effects including probiotic sepsis and no deaths. NEC≥Stage II occurred in only 1 neonate (placebo group).

**Conclusion:**

*B. breve* M-16V is a suitable probiotic strain for routine use in preterm neonates.

**Trial Registration:**

Australia New Zealand Clinical Trial Registry ACTRN 12609000374268

## Introduction

Necrotising enterocolitis (NEC) is a potentially serious and life threatening condition in preterm neonates [1], [2]. NEC occurs in 4–6% of very low birth weight (VLBW) neonates with significant mortality (20–25%) and morbidity including recurrent infections, protracted feed intolerance with dependence on parenteral nutrition, need for surgical intervention, and survival with short gut syndrome. The incidence (10–12%), mortality (40–45%), and morbidity of NEC including long term neurodevelopmental impairment after surgery for the illness, are higher in extremely preterm neonates [1], [2]. Considering its significant health burden, prevention of NEC is a priority. Prevention of prematurity, the single most important risk factor for NEC is difficult. Antenatal glucocorticoids, early preferential feeding with breast milk, prevention and treatment of infections, and standardised feeding protocols have been the important strategies available for prevention of NEC [3].

Probiotics are live microorganisms, which when administered in adequate amounts confer a health benefit on the host [4]. Their benefits include improved gut epithelial barrier function, enhanced mucosal IgA responses, increased production of anti-inflammatory cytokines, improved maturation of the immune system in the newborn, and suppression of pathogenic and promotion of beneficial microbes in the gut [5]. Despite decades of research, the pathogenesis of NEC remains unclear [1], [3]. Current understanding of the illness indicates a multifactorial pathophysiology, including excessive proinflammatory tendency of the immature gut, enteral feeding, and abnormal bacterial colonization with delayed diversity, and commensal richness of the gut flora [1], [3]. The rationale for probiotic prophylaxis relates to the role of bacterial colonisation of the gut in the pathogenesis of NEC [6]–[8]. Similar to earlier reviews [9], [10], results of a recent systematic review of randomised controlled trials (RCTs) confirm that probiotic supplementation significantly reduces the risk of NEC (RR: 0.33; 95% CI: 0.24–0.46; p<.00001), and all cause mortality (RR: 0.56; 95% CI, 0.43–0.73; p<.0001) without any significant adverse effects in preterm VLBW infants [11]. Considering the evidence in totality and in preparation for the introduction of, routine probiotic supplementation for preterm neonates in our unit, we performed this randomised trial [12]–[16]. The probiotic strain *Bifidobacterium breve* M-16V (Morinaga Milk Industry Co., Ltd, Japan) was selected based on previous studies of safety and efficacy with this strain and clinical experience with routine use in preterm neonates for over a decade in Japan [17]–[23]. Administration of this strain (1×10^9^ cfu twice daily) from birth until discharge has reduced clinical infection, sepsis, NEC and age to achieve full feeds in preterm neonates [17], [18]. In addition, with a dosage of 1.6×10^8^ colony forming units (cfu) twice daily, a more rapid development of the levels of bifidobacteria in stools during the first two weeks has been noted [19]. Approval for importing the product under the “Authorised prescriber pathway” (Therapeutic Goods Administration: TGA, Australia), and availability of the product and strain details from the manufacturer were the other reasons for selecting it.

## Hypotheses and Aim

We aimed to conduct a clinical trial to assess the effect of supplementation with *Bifidobacterium breve* (*B. breve*) M-16V on fecal bifidobacterium in preterm neonates. The hypothesis was that the supplementation will increase fecal *B. breve* counts.

The protocol for this trial and supporting CONSORT check list are available as supporting information; see Checklist S1 and Protocol S1.

## Participants and Methods

### Step 1: Laboratory assessment

An independent quality assessment of the probiotic product was conducted prior to the clinical trial to determine its suitability for use in preterm neonates. This involved the following steps: (1) Confirmation of strain species by molecular taxonomy methods (16S rRNA gene sequencing) [24]. (2) Checking for microbial contamination of the product in a TGA authorised laboratory (3) Checking osmolarity and stability (viable bacterial counts) of the reconstituted solution (4) Checking viability over 2 years after manufacturing and storing at temperature 22±2 degree Centigrade (5) Determining the antibiotic susceptibility profile by gradient diffusion (Etest, bioMérieux SA, Marcy l'Etoile, France.) [25]

### Step 2: Clinical trial

#### Design and setting

A randomised double blinded placebo controlled trial in preterm VLBW neonates admitted in our tertiary neonatal intensive care unit.

#### Eligibility criteria

(1) Gestation up to 32 weeks and 6 days (2) VLBW: Birth weight under 1500 grams (3) Ready to commence or on enteral feeds for <12 hours.

#### Exclusion criteria

(1) Major congenital malformation (2) Chromosomal aberration (3) Lack of informed parental consent (4) On enteral feeds for ≥12 hours (5) Contraindications for enteral feeds (6) Life threatening illness.

#### Outcomes

The primary outcome was the effect of *B. breve* M-16V supplementation on levels of *B. breve* in the stools of preterm neonates as detected by quantitative PCR. Secondary outcomes included evidence of a bifidogenic effect (elevation of total bifidobacteria in stools); incidence of NEC (≥Stage II) [26], and all cause death; time to reach full enteral feeds (150 ml/kg/day) and blood culture positive late onset sepsis (LOS) beyond 72 hours of life.

#### Safety

This was assessed by monitoring for (1) blood culture positive sepsis by *B. breve* M-16V and (2) adverse effects such as abdominal distension, vomiting, and diarrhea leading to cessation of the supplementation.

All outcomes and safety parameters were monitored from enrolment till death or reaching the corrected age of 37 weeks.

#### Pre-planned subgroup

A subgroup analyses was planned for extremely preterm neonates (Gestation <27 weeks) who are at the highest risk for mortality and morbidities, such as NEC, infections, and feed intolerance [1], [2].

#### Randomisation, allocation concealment, and blinding

Group assignment was allocated by a computer generated randomisation sequence in randomly ordered block sizes of 2 and 4, and stratified by gestational age at birth (up to 27^+6^ weeks and ≥28 weeks) to ensure that extremely preterm neonates were equally distributed between the two arms of the trial. Opaque, sealed, coded envelopes were used for randomisation. Neonates of multiple pregnancies were considered as separate individuals. Allocation concealment was optimised by prescribing allocation only after informed parental consent and recording the basic neonatal data. The Clinical Trial Pharmacist (CTP) supplied the randomisation sequence and the sachets (identical design, weight, smell, and taste) containing either the probiotic (*B. breve* M-16V; 5×10^9^ cfu per sachet with dextrin as carrier) or placebo (equal volume of dextrin) manufactured by Morinaga Milk Industry Co., Ltd, Japan, to the nursing staff. This assured masking of all investigators, clinical and non-clinical outcome assessors, nursing staff and parents with regards to the allocation status of enrolled neonates.

#### Probiotic protocol

When ready for enteral feeds, enrolled neonates were supplemented with the freshly reconstituted contents of the allocated sachets every day, and continued until the corrected age 37 weeks. Reconstitution of the dry powder in the sachets was done using sterile water for injection or breast milk when available. Care was taken during reconstitution to reduce the risk of cross contamination by adhering to strict hand hygiene, preparing doses for individual neonates separately, and avoiding contact with indwelling lines, tubes, and catheters. The dose was 3×10^9^ cfu/day (1.5 mls of the reconstituted solution), given as a single dose via the orogastric feeding tube. The dose and duration of supplement was based on the previous clinical, and experimental (oral toxicity) studies of this strain in preterm neonates [17]–[19], [22]. For neonates ≤27 weeks the daily dose was 1.5×10^9^ cfu per day until reaching milk feeds of 50 ml per kg per day. It was then increased to 3×10^9^ cfu per day. Considering the risk of probiotic sepsis, supplementation was stopped when enteral feeds were stopped by the attending neonatologist for indications such as sepsis and NEC.

The manufacturer Morinaga Milk Industry Co., Ltd, Japan was not the sponsor but only supplied the product free for the trial.

#### Stool samples

Two stool samples were collected for quantitative cultures from each neonate: One before and one 3 weeks after starting the probiotic supplementation. Samples were frozen after collection and stored at −80 degree Centigrade prior to analysis. The investigators (PC, ME) involved in stool culture studies were masked to the allocation status of the enrolled neonate, assuring masking of the primary outcome assessor.

Stool cultures: The stool samples were thawed on ice prior to analysis. Stool samples with very inadequate volume were not analysed. The total viable bifidobacteria were enumerated in triplicate by 10-fold serially diluting samples in Wilkins Chalgren broth and plating aliquots on Reinforced Clostridial Agar supplemented with aniline blue (0.03%) as previously described [27]. Plates were incubated at 37 C for 48 hours. The aniline blue and propionic acid in this medium were selective for the bifidobacteria. Pale blue colonies were presumptively identified as bifidobacteria. Results of the total viable bifidobacteria were expressed as cfu per gram (cfu.g^−1^). The *B. breve* was enumerated by quantitative PCR of DNA extracted from the stool samples according to the method of Matsuki et al (2003) [28]. Briefly, the DNA was released from washed cell suspensions using lysate buffer (100 mM Tris-HCl, 40 mM EDTA, 1% SDS, pH 9.0), 0.1 mm glass beads and a bead beater and then treated with phenol-chloroform-isoamyl alcohol (25∶24∶1) prior to precipitation with 3M sodium acetate in 95% ethanol. The *B. breve* specific primer set (BiBRE-1 CCGGATGCTCCATCACAC and BiBRE-2 ACAAAGTGCCTTGCTCCCT) was used, and in order to enhance specificity, real time PCR conditions were optimised using SsoFast Evagreen (BioRad) as the DNA binding dye instead of SYBR green as used by Matsuki et al [29]. A *B. breve* M-16V strain-specific-primer reported by Schouten et al (2009) was not used due to the potential for cross amplification of *B. breve* other than the M-16V strain [30].The amplification consisted of a cycle at 98°C for 2 min, 40 cycles of 20 secs at 95°C then 63°C, 72°C for 30 secs, 83°C for 20 secs followed by analysis of melt curves from 65 to 95°C.

#### Sample size

Sample sizes of 50 per group were estimated to achieve approximately 90% power to detect the colonisation rate of 30% in the probiotic versus 5% in the control group when using a two-sided test of proportions with continuity correction at 5% significance level. Initially, an additional 20 neonates (10 per arm) were estimated to cover for the loss to follow up. However, in the early phase of the study, we realised that there were difficulties in collecting timely stool samples, and the volume of stool sample was frequently too small for analysis. Hence instead of 10 per arm, an additional 30 neonates per arm were enrolled. This increase also allowed us to specifically study the effect of probiotic supplementation on innate immunity in preterm neonates by comparing results between those randomised to either probiotic or placebo. These will be presented in a separate publication.

#### Ethics statement

Approval was obtained from the Women and Newborn Health Services (WNHS) Ethics Committee at KEM Hospital for Women, Government of Western Australia, Department of Health, Perth. Ethics approved written informed consent was obtained from the parents before enrolling neonates in the trial. Clinical Trials Notification approval was obtained from TGA, Australia.

#### Trial registration

The trial protocol was registered under the Australian Clinical Trials Registry (ACTRN 12609000374268).

#### Statistical methods

The analysis was based on the intention to treat principle. Group outcomes were summarised using medians, interquartile ranges and ranges for continuous outcomes. Categorical outcomes were summarised using frequency distributions. Univariate comparisons for continuous data were made using Mann Whitney tests and for categorical data using Chi-square or Fisher exact tests. Comparisons of pre and post colonisation frequencies and counts were made using the McNemar test and the Wilcoxon signed-rank test. Colonisation counts below the detection limit were assigned a value of 27,500 cfu/g (halfway between 0 and the detection limit of 55,000 cfu/g), and all values were transformed to the base 10 logarithm for analysis. A pre-planned subgroup analysis was conducted on extremely preterm (gestation <27 weeks) neonates, and probiotic counts, sepsis and feeding outcomes were assessed. A Bonferroni adjustment was applied for each outcome reported in the subgroup analysis, such that the significance level for each comparison of probiotic vs placebo groups within gestational age strata was set to 0.025. All tests were two-sided, and a p-value<0.05 was considered statistically significant for the primary analysis. The analysis was performed using SPSS 18.0 for Windows (SPSS, Chicago, IL) and StatXact 8.0, Cytel Inc, 2007.

#### Data handling, storage, confidentiality

The NHMRC Australian guidelines were followed for confidentiality and data storage [31].

#### Reporting

The revised CONSORT guidelines were used for reporting the trial results [32].

## Results

### Laboratory assessment

Results of the independent quality assessment supported the data provided by the manufacturer. The product did not have any microbial contaminants; viability was maintained on storage at 22±2 degree Centigrade for 2 years after the manufacturing date. The osmolarity of the reconstituted solution (with breast milk) was 320–350 mOsm/L, which is reported to be safe for VLBW neonates [33]. 16S rRNA gene sequencing confirmed *B. breve* species identity with a match of 1408/1410 nucleotides (99.86%) with *B. breve* strain ATCC 15700 (GenBank: AB006658.1). The strain was susceptible to the antibiotics commonly used for early and LOS in our nursery including penicillin, cefotaxime, ceftriaxone, meropenem, and vancomycin.

### Clinical trial

**Figure 1:**
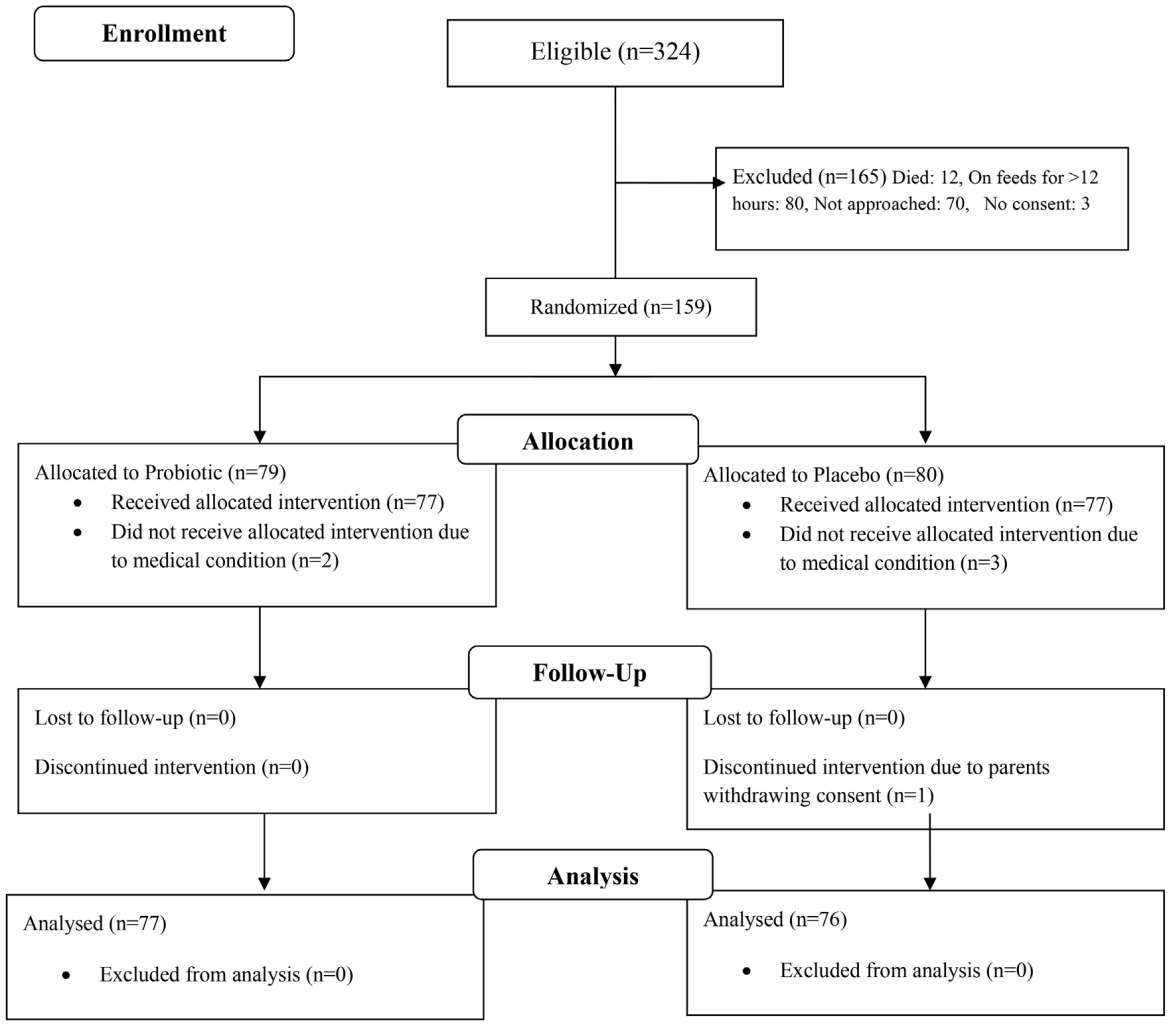
CONSORT Flow Diagram.

A total of 159 neonates (Probiotic: 79, Placebo: 80) were enrolled in the trial between November 2010 and May 2012 (Figure 1). Two neonates in the probiotic group did not receive the allocated supplementation due to medical condition. Three neonates in the placebo group did not receive treatment allocation due to medical condition, and in another case the parents withdrew the consent before completion of treatment. The final analysis thus included 77 and 76 neonates in the probiotic and placebo group, respectively. There was no significant difference in maternal and neonatal demographic characteristics (Table 1).

**Table 1:** Maternal and infant birth characteristics.

	Probiotic N = 77	Placebo N = 76
Maternal characteristics		
Maternal PIH	22 (29%)	15 (20%)
Maternal APH	20 (26%)	14 (19%)
Chorioamnionitis	9 (12%)	11 (15%)
PPROM >24 hours	16 (21%)	23 (31%)
Maternal antibiotics	65 (84%)	65 (86%)
Antenatal glucocorticoids		
Complete	33 (43%)	39 (51%)
Incomplete	35 (46%)	28 (37%)
None	9 (12%)	9 (12%)
Inborn	75 (97%)	72 (95%)
Cesarean section	58 (75%)	49 (65%)
Gestation (weeks)	29 (26–30; 23–32)	28 (26–29; 23–33)
Gestation ≤27 weeks	28 (36%)	29 (38%)
Neonatal characteristics		
Birth weight (grams)	1090 (755–1280; 466–1830)	1025 (810–1260; 480–1770)
Male	45 (58%)	41 (54%)
SGA	25 (33%)	25 (33%)
Apgar <7 at 5 minutes	14 (18%)	19 (25%)
CRIB score	6 (3–9; 1–16)	7 (4–10; 1–16)
PDA	31 (40%)	34 (45%)
NBM during treatment for PDA#	9 (31%)	19 (56%)
Feed type at reaching full feeds (150ml/kg/day)		
EBM	68 (88%)	64 (84%)
PDHM	18 (23%)	17 (22%)
PTF	1 (1.3%)	-

Data represents median, 25^th^–75^th^ percentile and minimum-maximum or N (%), as appropriate.

PIH: Pregnancy induced hypertension, APH: Antepartum haemorrhage.

PPROM: Preterm prolonged rupture of membranes, SGA: Small for gestational age.

CRIB score: The CRIB (clinical risk index for babies) score: a tool for assessing initial neonatal risk and comparing performance of neonatal intensive care units. The International Neonatal Network. [No authors listed] Lancet 1993 Jul 24;342(8865):193–8. Erratum in: Lancet 1993 Sep 4;342(8871):626.

NBM: Nil by mouth. PDA: Patent ductus arteriosus, EBM: Expressed breast milk, PDHM: Pasteurised donor human milk, PTF: Preterm formula.

# p-value = 0.029.

### Primary outcome (*B. breve* counts in stool samples)

A total of 74 neonates in the probiotic group, and 66 in the placebo group had the two stool samples (before and 3 weeks after the supplement; referred to as sample 1 and 2 respectively) available for analysis (Table 2). There were 24 (31%) and 22 (29%) neonates in the probiotic and placebo groups respectively who commenced their probiotic or placebo treatment prior to the first stool collection (p = 0.805). The proportion of neonates with detectable *B. breve* increased in both the placebo and probiotic groups after the intervention: Placebo: 2/66 (3%) in sample 1 to 25/66 (38%) in sample 2, p<0.001 and Probiotic: 29/74 (39%) in sample 1 to 67/74 (91%) in sample 2, p<0.001. Of the 29 neonates in the probiotic group with counts above the detection limit for the first stool sample, 15 (52%) had commenced treatment prior to the first collection.

**Table 2:** Primary and secondary outcomes.

Outcome	Probiotic N = 74	Placebo N = 66	p-value*
Primary outcome			
*Bifidobacterium breve* (cfu.g-1)			
Sample 1			
Count (log_10_) ***Median (75^th^percentile-maximum)***	BD (6.7–9.1)	BD (BD-8.0)	<0.001
Colonised	29 (39%)#	2 (3%)	<0.001
Sample 2			
Count (log_10_) ***Median (75^th^percentile-maximum)***	8.6 (8.9–9.4)	BD (7.6–9.5)	<0.001
Colonised	67 (91%)	25 (38%)	<0.001
Difference (S2-S1) (log_10_) *Median (75^th^percentile-maximum)*	3.1 (4.3–4.9)	0 (3.2–5.0)	<0.001

Secondary outcomes	N = 77	N = 76	
All cause deaths	-	-	
NEC≥Stage II	-	1	0.497
Late onset sepsis	17 (22%)	12 (16%)	0.410
Time to reach full enteral feeds (150 ml/kg/d) in days	12 (9–21;5–71)	12 (8–16; 3–81)	0.306
Length of hospital stay (w)	10 (6–14; 2–61)	10 (7–14; 3–60)	0.812
Discharge weight (g)	2590 (2184–2990; 1565–4290)	2565 (2303–3080; 1605–5074)	0.539
**Early onset sepsis**			
Suspected	77 (100%)	74 (98%)	0.245
Proven	4 (5%)	2 (3%)	0.681
Duration antibiotics (d)	3 (3–5; 2–14)	3 (3–5; 3–18)	0.685
**Late onset sepsis**			
Suspected episodes			
None	48 (62%)	43 (57%)	0.744
1	15 (20%)	16 (21%)	
2+	14 (18%)	17 (22%)	
**Proven episodes**			
None	60 (78%)	64 (84%)	0.465
1	12 (16%)	10 (13%)	
2+	5 (7%)	2 (3%)	
**Duration antibiotics**	7 (5–10; 3–21)	6 (3–11; 2–33)	0.296

Data represents median, 25^th^–75^th^ percentile and minimum-maximum or N (%), unless otherwise stated.

BD = below detection limit. Counts below the detection limit were assigned the value 4.4 [log_10_(27,500)] for analysis.

# 15/29 (52%) neonates had commenced probiotic treatment prior to the first collection.

S2-S1: Difference between sample 2 and sample 1.

*Chi- square test was used to generate p values.

Sample 1 and 2 *B. breve* counts were higher in the probiotic group than the placebo group. The median counts in both groups were below detection (<4.7 log cfu per g) in sample 1, and significantly increased in sample 2 for the probiotic group (8.6 log while remaining <4.7 log in the placebo group. The difference (sample 2-sample 1) was significantly higher in the probiotic group than the placebo group (p<0.001).

### Subgroup analysis

For neonates with gestation ≤27 weeks, sample 1 and 2 counts were significantly higher in the probiotic versus placebo group (Table 3). The rise in counts from sample 1 to 2 was significantly higher in the probiotic group whereas the median sample 1 and 2 counts remained below the limit of detection (LOD) in the placebo group. For neonates with gestation >27 weeks sample 1 counts were below the LOD in the probiotic as well as the placebo group. The median counts increased significantly in sample 2 in both the probiotic and the placebo group neonates. There was no significant difference in the frequency of LOS, and the time to reach feeds of 150 ml/kg/day in the probiotic versus placebo group neonates within gestation subgroups ≤27 versus >27 weeks.

**Table 3:** Primary and secondary outcomes stratified by gestational age at birth.

Outcome	≤27 weeks			>27 weeks		
Primary outcome	Probiotic N = 27	Placebo N = 27	p-value*	Probiotic N = 47	Placebo N = 39	p-value
*Bifidobacterium breve* (cfu.g-1)						
Sample 1						
Count (log_10_) ***Median (75^th^percentile-maximum)***	5.2 (7.9–8.9)	BD (BD-5.2)	<0.001	BD (6.2–9.1)	BD (BD-8.0)	0.001
Colonised	14 (52%)	1 (4%)	<0.001	15 (32%)	1 (3%)	<0.001
Sample 2						
Count (log_10_) ***Median (75^th^percentile-maximum)***	8.6 (8.9–9.1)	BD (BD-9.5)	<0.001	8.6 (8.9–9.4)	5.5 (8.2–9.3)	<0.001
Colonised	25 (93%)	5 (19%)	<0.001	42 (89%)	20 (51%)	<0.001
Difference (S2-S1) (log_10_) *Median (75^th^percentile-maximum)*	2.9 (4.4–4.7)	0 (0–5.0)	0.010	3.2 (4.3–4.9)	1.1 (3.8–4.9)	0.007

Secondary outcomes	N = 28	N = 29		N = 49	N = 47	
Late onset sepsis	11 (41%)	6 (21%)	0.248	5 (10%)	5 (11%)	1.000
Time to reach full enteral feeds (150 ml/kg/d) in days	21 (12–29;8–71)	14 (12–20;7–47)	0.073	10 (7–15; 5–38)	10 (7–13;3–81)	0.672

Data represents median, 25^th^–75^th^ percentile and minimum-maximum or N (%), unless otherwise stated.

BD = below detection limit.

Counts below the detection limit were assigned the value 4.4 [log_10_(27,500)] for analysis.

p-values<0.025 considered significant after Bonferroni adjustment.

S2-S1: Difference between sample 2 and sample 1.

*Chi- square test was used to generate p values.

### Secondary outcome (*Bifidobacteria* counts in stool samples)

For the enumeration of the total bifidobacteria in the samples, there were 42 placebo and 46 probiotic subjects with no detectable bifidobacteria in the initial samples (sample 1); 4 placebo and 3 probiotic subjects had no detectable bifidobacteria in the second sample (sample 2). There was no significant difference in the total bifidobacteria count in the final samples (sample 2) when the probiotic (8.6 log cfu.g^−1^) and placebo (8.4 log cfu.g^−1^) groups were compared.

### Secondary (Clinical) outcomes

Neonatal clinical characteristics did not differ between groups, except for a higher proportion of placebo group neonates who were nil by mouth during treatment for patent ductus arteriosus (Table 1). The number of episodes of early and late onset suspected or proven (blood culture positive) sepsis was not different between the two groups (Table 2); the nutrition and feeding related characteristics also did not differ between groups as there were similar rates of breast feeding in the two group ie 84 and 81% probiotic and control, respectively, P>0.4 across the entire duration of the study. Over the first 12 days rates averaged 89 and 87% for probiotic and control, respectively.

Time to reach full feeds was 12 days (9–21;5–71) for the probiotic group and 12 days (8–16;3–81) for the placebo group (p = 0.306) (Table 2). There were no deaths in either the probiotic or the placebo group neonates. There was only one case of NEC≥Stage II in the placebo group.

**Safety:** There was no case of blood culture positive sepsis by *B. breve* M-16V. There were no adverse effects such as abdominal distension, vomiting, and diarrhea leading to cessation of the supplementation in any of the enroled neonates.

## Discussion

Our results indicate that *B. breve* M-16V supplementation is safe, and effective in enhancing levels of *B. breve* in stools of preterm VLBW neonates. While we were not able to confirm identification of the M-16V strain in the stools due to the potential for PCR amplification primers detecting other *B. breve* strains, the significantly elevated levels of *B. breve* species in the probiotic treated group is strong evidence suggesting that the M-16V strain was present in the stools of these neonates. This is further supported by the fact that there were no significant differences in the number of placebo (control) or probiotic dosed neonates with detectable bifidobacteria (total viable bifidobacteria; any species) or the level of bifidobacteria after administration of the M-16V strain, yet the number of *B. breve* was significantly greater in those receiving the probiotic. The levels of *B. breve* were 8.6 log and <4.7 log in the probiotic and placebo groups, respectively while total bifidobacteria (all species) was 8.6 and 8.4 log in the probiotic and placebo groups, respectively. This implies that the bifidobacteria in the probiotic dosed group were predominantly *B. breve*, while those in the placebo group were not. Our findings that 38% and 91% of placebo and probiotic dosed neonates, respectively, are colonised with *B. breve* at three weeks are consistent with those of Kitajima et al (1997) who detected *B. breve* (Yakult strain) in 12% and 73% at 2 weeks and 28% and 82% at 4 weeks, in control and probiotic dosed groups, respectively [34].

*B. breve* is one of the most frequently isolated species of *Bifidobacterium* in one month old healthy breast fed infants (Mikami et al, 2012) [35], and is also common in breast milk (Martin et al, 2009) [36]. Consequently, elevating fecal *B. breve* levels in preterm neonates could be considered a positive outcome. It is acknowledged that breast fed infants have a lower incidence of respiratory, digestive and immunological related diseases and hence elevating fecal *B. breve* levels could be considered to have a potential health benefit. Consequently it is postulated that since dosage with *B. breve* M 16V elevated *B. breve* levels, health benefits could be anticipated.

Considering that *B. breve* is one of the species frequently found in intestinal tracts of healthy infants in the first month of life [29], [35] and in breast milk [36], its presence in stools of some of the placebo recipients is expected. Additionally, despite all care the possibility of cross contamination of control group neonates can not be excluded.

It is important to note that the median value for the total bifidobacteria in the stools was greater in the probiotic group (n = 74) after 3 weeks of supplementation, but the difference was not significant when compared to the placebo group (n = 66). Only 4% of the probiotic supplemented neonates and 6% of placebo neonates had no detectable bifidobacteria after the 3 weeks while Li et al [19] reported 80% of the control LBW neonates had no detectable bifidobacteria, albeit with only 10 subjects in the group. The higher level of bifidobacteria colonisation in the placebo controls in our study could be attributed to many compounding factors including greater usage of breast milk. Unfortunately Li et al [19] did not publish the breast feeding rates to allow comparison with the high rate of 89% in our study. Furthermore one can suppose that if the breast feeding rate had been lower in our study, greater differences would have been observed.

The higher *B. breve* counts in sample 1 can be explained by the fact that our protocol was to start the study supplement as soon as the neonate was ready for feeds, and not to wait for the passage of meconium which is very unpredictable, especially in extremely preterm (gestation <27 weeks) neonates who are at the highest risk of NEC. We hence decided not to delay the supplement if the neonate was otherwise well enough for starting enteral feeds. This could be considered as one of the limitations of our study.

The supplement was well tolerated by all enrolled neonates with no adverse effects such as abdominal distension, diarrhea, or vomiting; despite the slightly higher dosage (up to 3×10^9^ cfu daily vs. 1×10^9^ cfu once/twice daily) than previously reported [17], [18]. Our independent assessment confirms the quality of the product/strain as claimed by the manufacturer.

It is important to note that our trial was not powered to detect improvement in NEC, and/or all cause mortality as primary outcomes. A conservative sample estimate would require in excess of 5,000 neonates to assess if probiotic supplementation reduces the risk of these outcomes considering their low baseline incidence in our unit.

The effects of probiotics are strain specific, hence noting the results of previous studies of this specific probiotic strain in preterm neonates is important [17]–[23]. Umezaki et al and Satoh et al have reported the benefits of this strain in preterm ELBW and VLBW neonates in presence of low baseline incidence of NEC and sepsis [17], [18]. Li et al have reported the beneficial effects of *B. breve* M-16V on gut flora in LBW infants [19]. Other workers have studied effects of *B. breve* dosage on metabolites e.g. short chain fatty acids (SCFAs) in the gut. While SCFAs provide energy to colonocytes, their overproduction may cause mucosal injury in preterm infants [37], [38]. Wang et al reported that *B. breve* reduces the production of butyric acid, which may provide protection from NEC [20].Wang et al reported that *B. breve* reduces the production of butyric acid, which may provide protection from NEC [20]. Transforming growth factor (TGF) A1 displays a broad spectrum of activities in mucosal regulation, including induction of oral tolerance, potent anti-inflammatory effects, mucosal IgA expression and effects on epithelial cell proliferation and differentiation [39]. Fujii et al reported that *B. breve* M-16V can up-regulate TGF-A1 signaling and may be beneficial in attenuating inflammatory and allergic reactions in preterm infants [21].The single dose and 90-day repeated dose oral toxicity tests in rats (Abe et al 2009) indicate that the test strains had no translocation ability and induced no damage to intestinal surface [23].

In summary, our results, together with data from previous studies confirm that the probiotic strain *B. breve* M-16V is suitable for use in preterm neonates. Despite the assuring strain specific data, careful surveillance is necessary for early detection and management of adverse effects, including probiotic sepsis, as their risk is not zero for any live probiotic strain when used in an immunocompromised population [40], [41].

## Supporting Information

Protocol S1 **Trial protocol.**
(DOC)

Checklist S1 **CONSORT Checklist.**
(DOC)

Ethics Approval S1 (DOC)
