# Supplementary material for: Effect of Bifidobacterium breve M-16V Supplementation on Fecal Bifidobacteria in Preterm Neonates - A Randomised Double Blind Placebo Controlled Trial
Source: PLoS One. 2014 Mar 3;9(3):e89511. doi: 10.1371/journal.pone.0089511 (PMC3940439; doi:10.1371/journal.pone.0089511)

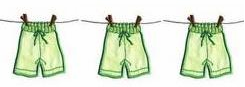


**Protocol Flow Sheet**

**Recruitment:**

- Review inclusion/exclusion criteria
  - Inclusion criteria:
    - Gestation <33 weeks
    - Birthweight <1500g
    - Has not been on feeds for >12hours
  - Exclusion Criteria:
    - Major congenital malformation
    - Chromosomal aberration
    - Lack of parental consent
    - On enteral feeds ≥12 hours
    - Contraindication for enteral feeds
    - Life threatening illness / condition
- Obtain parental consent
- Original in PANTS record
- Signed copy to parent
- Signed copy to infant’s hospital record

**Day 1**

- Stool sample to be collected from every baby fitting inclusion criteria
- To be labeled with patient sticker with date and time
- Sample to be stored in WIRF freezer
  - If not consented discard sample
  - If consent obtained assign study number and remove old UMRN sticker

Out of hours: Stool sample to be stored in the PANTS fridge in SCN gas room.

- Flag infant as in study in:
  - Coordinator’s handover folder
  - Inpatient notes (copy of consent)
  - Patient clip board
  - Study Log

**Randomisation:**

- Eligible infants, with parental consent, will be randomised into either Group A or Group B
- Randomisation will be done by the research nurse

**Administration of Study Supplement (probiotic/placebo)**

- Charted by Drs in drug chart
- Double checked by two nurses (like any other drug)
- Supplement is given between feeds
- Dose:
  - **Minimal enteral feeds (<50ml/kg/day)**
    - 1.5ml of reconstituted supplement once per day
  - **Nutritive enteral feeds (>50ml/kg/day)**:
    - 1.5ml of reconstituted supplement twice a day
  - Example:

Baby is on 0.5ml EBM 6 hourly, supplement dose is 1.5mls daily, given between feeds

- - See Appendix I for sample drug chart record
- Reconstitution & Administration

1. Take sachet from correct patients bag in drug fridge
2. Draw up 4mls of Water for Injection in 10ml syringe
3. Withdraw plunger to top of syringe and screw red combi-stop onto end
4. Remove plunger
5. Cut top off sachet and empty entire contents of sachet into syringe
6. Replace plunger and shake well until totally dissolved (will be a milky colour). 1ml = 1.5 billion organisms
7. Discard 2.5mls (leaving 1.5ml solution)
8. Test OGT/NGT placement as per unit protocol
9. Give remaining 1.5ml (1.5 billion organisms) via OGT/NGT
10. Document “PANTS supplement 1.5mls” in comments section of obs chart
11. Sign drug chart

- The baby will continue on the supplement until 37weeks or discharge, which ever comes first.

**Day 21:**

- Collect stool sample and store in WIRF freezer
- Document on:
  - data sheet
  - study log
  - sticker on patients clip board

**Discharge / Baby 37weeks:**

- - Cease supplement on drug chart
  - Flag infant as completed study in:
- coordinator’s handover folder
- clip board sticker
- study log
- Complete data entry
  - Thank parents & staff

**If Baby Transferred:**

- Ensure research nurse is aware of transfer
- Education to staff at peripheral hospital
  - Study Information
  - Administration
  - Reporting SAE / AE
- Ensure adequate supply of product at site
- Ensure appropriate storage of product at site
- Follow up baby at site

**Safety:**

- - If enteral feeds cease – withhold the study supplement until consultant review
  - If positive blood culture from probiotic organism - withhold the study medication until consultant review and report to DSMB
  - Report SAE / AE to principal investigator
  - Report to safety committee

**Un-blinding:**

- In the event of subject requiring to be un-blinded
  - Contact principle investigator (Dr Patole)
  - Contact Antonia Wong in pharmacy who will have randomisation schedule
  - Complete data entry
  - Communicate events to parents

**Sample Collection:**

- Stool will be collected in supplied container, sample size of a walnut if possible. Ensure the date and time of collection is noted on the sample label.
- Sample will be stored at -80ºC in the WIRF freezer space
  - Transported to freezer in esky containing ice or freezer block
- Samples collected out of hours will be stored in PANTS fridge in the SCN gas room until can be transferred to freezer
- Midway through, and at the end of the study, the samples will be transported by DMG couriers on dry ice to Professor Conway at the University of NSW, Sydney.

**Contact people:**

- Principal Investigator: Dr Sanjay Patole – 9340 1260
- Research Nurse: Alessia Chiera – pg 1717, ph 0438 988 249

Neonate admitted:

GA < 33 weeks **&**

BW < 1500g

Collect first stool passed

If in office hours research nurse to label and take directly to freezer

If is in after hours, sample to be stored in fridge in SCN3 gas room

Obtain informed consent from parents

If consent obtained and has not been on feeds for >12 hours enroll baby in the study and assign a study number

Parents decline consent

Randomise into group A or B

Dr to chart supplement in drug chart

Stool sample discarded

- Document details in data sheet

- Copy of consent into pt notes

- Copy of consent to parents

- Trial ID sticker on pt clip board & Handover file

Obtain stool sample after 21 days of supplement

- Cease supplement at 37wk or discharge

- Complete data log

- Thanks parents

**Appendix I**

1A. Example medication chart – **Minimal enteral feeds**

| Date  Ordered | Drug  **PANTS TRIAL SUPPLEMENT** | | | |
| --- | --- | --- | --- | --- |
| Pharmacist | First Dose | Route | Special Directions  **As per PANTS Protocol** | |
|  | Next Doses  **1.5ml** | Route  **ORAL** | Frequency  **daily** | Duration  **Until >50mg/kg/day**  **feeds** |
| Date  Canc’ld | Dr’s Name PRINT  Dr’s Signature | | | |

1B. Example medication chart – **Nutritional enteral feeds**

| Date  Ordered | Drug  **PANTS TRIAL SUPPLEMENT** | | | |
| --- | --- | --- | --- | --- |
| Pharmacist | First Dose | Route | Special Directions  **As per PANTS Protocol** | |
|  | Next Doses  **1.5ml** | Route  **ORAL** | Frequency  **12 hrly** | Duration  **Until 37weeks** |
| Date  Canc’ld | Dr’s Name PRINT  Dr’s Signature | | | |

**Appendix II**

**Reconstituting PANTS supplement**

1. Take sachet from correct patients bag in drug fridge
2. Draw up **4mls** of Water for Injection in 10ml syringe
3. Withdraw plunger to top of syringe (ie 12ml mark) and screw red

combi-stop onto end

1. Remove plunger
2. Cut top off sachet and empty entire contents of sachet into syringe
3. Replace plunger and shake well until totally dissolved (will be a milky colour). 1ml = 1 billion organisms
4. Give **1.5ml** (1.5 billion organisms) to patient [discard remainder of solution]


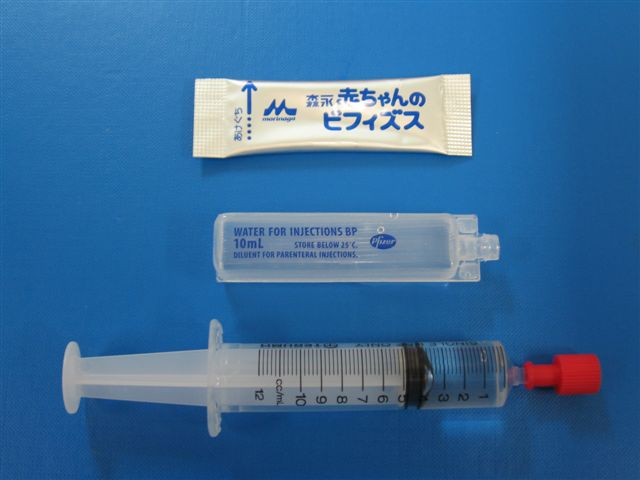

Supplement: Protocol S1 — Trial protocol. (DOC) [file pone.0089511.s001.doc]
