# Supplementary material for: Effect of Bifidobacterium breve M-16V Supplementation on Fecal Bifidobacteria in Preterm Neonates - A Randomised Double Blind Placebo Controlled Trial
Source: PLoS One. 2014 Mar 3;9(3):e89511. doi: 10.1371/journal.pone.0089511 (PMC3940439; doi:10.1371/journal.pone.0089511)
Supplement: Ethics Approval S1 — (DOC) [file pone.0089511.s003.doc]

**Friendly bacteria to save premature babies from death and disease**

Sanjay Patole^1,4^, Tony Keil ^2^, Dorota Doherty^3,4^, Karen Simmer^1,4^, Patricia Conway^5^

Departments of Neonatal Paediatrics^1^, Department of Microbiology^2^ and Women and Infants Research Foundation^3^ at KEM Hospital for Women, University of Western Australia^4^, Perth, University of New South Wales^5^, Sydney

*Short title: Probiotics for reducing death and disease in premature babies*

**Trial registration: ACTRN 12609000374268**

Registered: 27/5/2009

**Correspondence**

Prof Sanjay Patole, MD, DCH, FRACP, MSc, DrPH

Department of Neonatal Paediatrics, KEM Hospital for Women

374 Bagot Road, Subiaco, Perth, Western Australia 6008

Tel: 08-93401260, Fax: 08-93401266

E-mail: [sanjay.patole@health.wa.gov.au](mailto:sven.schulzke@health.wa.gov.au%0CAbstract)

PROJECT SUMMARY

Probiotics are the live beneficial microorganisms that are naturally present in the digestive tract.^1-4^ These friendly bacteria promote health by suppressing the growth of potentially harmful bacteria, improving immune function, enhancing the protective barrier of the digestive tract, and helping us in producing vitamin K. Human beings have been consuming these friendly bacteria for hundreds of years in the form of various food supplements, the commonest being Yogurt, and “Yakult” (containing the Shirota strain of the friendly bacteria lactobacillus casei). Death and diseases like necrotising enterocolitis (NEC- a condition with a gangrenous bowel), infections, and feeding difficulties due to immature bowel function are a major problem in premature babies worldwide. Many clinical trials have evaluated the safety and benefits of probiotic supplementation in premature babies. Meta-analysis of the results of clinical trials is an advanced statistical overview that provides the highest quality evidence to guide clinical practice. Our team at KEM Hospital for Women, Perth, conducted such an overview of 7 trials involving 1393 premature babies born under 33 weeks with birth weight under 1.5 kg.^5^ The results of this overview published in one of the most respected medical journals, Pediatrics The Lancet, showed that the risk of death and NEC was reduced by 53% and 64% respectively in babies receiving probiotic supplement compared with control group babies. The time to achieve full milk feeds was also significantly less (by an average of nearly three days) in babies receiving probiotic supplement. ^5^ These remarkable, consistent, and reliable results indicated the tremendous potential of probiotic supplementation in saving premature babies from death and disease. Experts had commented that the overview by our team was an important step towards evidence-based use of probiotics in premature babies. Subsequent overviews had confirmed that the evidence supported routine use of probiotic supplements in very low birth weight premature babies.^6,7^ *Recently 4 more trials have reported the safety and benefits of probiotic supplementation in premature babies.^8^ Our recent conclusive overview of these 11 trials has again confirmed the benefits of probiotics in reducing the risk of death and NEC in premature babies.^8^* Researchers in Japan have already been using probiotic supplementation as a routine for very (birth weight under 1500 grams) as well as extremely (Birth weight under 1000 grams) low birth weight babies since 1999.^9^ Probiotics have also been shown specifically to improve feed tolerance, bowel habits, and GI motility in premature babies.^10^ KEM Hospital for Women is the sole tertiary neonatal referral centre for the State of Western Australia. Every year this extremely busy unit admits ~500 extremely premature babies who are at risk of death and diseases like NEC. Given the current high quality evidence, the benefits of continued research on supplementation with friendly bacteria in this high-risk population can not be over estimated. There are significant regulatory and technical difficulties in accessing a proven, safe, and effective probiotic product for routine use in premature babies. Our proposed two step research involves rigourous evaluation of the safety and efficacy of a probiotic product before selecting it for subsequent routine use in premature babies. Step one is laboratory testing of the product to confim its contents (taxonomy confirmation) and safety (e.g. contaminents, unspecified components, osmotic load, pH). Step two is a pilot clinical trial of the product to evaluate its ability to colonise the gut and tolerance in premature babies and risk of infections due to the probiotic organisms.

**Budget**

[1] **Handling of stool specimen** at KEMH (receipt, labelling, storage, maintaining log book, and transfer to Sydney for processing) at **$50 per specimen** plus **Quantitative cultures on stool samples** in Prof Patricia Conway’s laboratory in Sydney at **$150 per specimen. Total $ 200 per stool specimen. (Total cost for 200 samples from 100 babies= $ 40000)**

[3] **Independent taxonomy confirmation** (species specific PCR) of the probiotic strains in the Department of Microbiology at KEMHW (Dr Tony Kiel): **$ 5,000**

**[4] Probiotic supply cost:** **$ 5,000**

**[5] Research assistant for coordinating the study** (ongoing education sessions, obtaining informed parental consent, monitoring sample collection, storage and transfer and collecting laboratory results, collecting demographic and clinical outcome data, organising data for analysis, and helping the principle investigator in preparing the manuscript for review toward publication): **Salary $ 20,000** (Based on full time salary ~$ 32375 per year as per current guidelines for Professional Research Person (PSP3).

**GRAND TOTAL: $70,000 (For 100 babies enrolled over ~6-8 months)**

**Salary:**

If 38 hours per fortnight x 4 weeks @ 36.91970 per hour = $5611.79 gross. (Non shift worker)

**PROJECT DETAILS**

**HYPOTHESES AND AIM**

We aim to conduct (1) independent microbiological studies to confirm the identity of the probiotic bacteria and the safety (contaminants, unspecified contents, osmolarity) of the probiotic product followed by (2) a pilot clinical trial to test the hypothesis that routine supplementation with this probiotic product will result in colonisation of the gut (primary hypothesis) and reduction in all cause deaths, and diseases like NEC, and late onset sepsis (secondary hypotheses) in premature babies.

**PARTICIPANTS AND METHODS**

**STEP 1 (Independent laboratory testing of the probiotic product)**

16S rRNA gene sequencing technique would be used to confirm the taxonomy of the probiotic organisms.^11^ The product would also be tested for contaminants, unspecified organisms, and osmolarity to determine its suitability for premature babies.

**STEP 2 (Pilot clinical trial of the probiotic product)**

**Design and setting:** This will be a randomised double masked placebo controlled pilot trial in premature very low birth weight (VLBW) babies admitted in our tertiary neonatal intensive care unit. A placebo controlled RCT design is selected to minimise bias in evaluating the safety and efficacy of the probiotic bacteria and in generating reliable data prior to their routine use in our nursery.

## Eligibility criteria: (1) Gestation up to 32 weeks and 6 days (2) VLBW: Birth weight under 1500 grams (3) Ready to commence or on enteral feeds for up to 12 hours

**Exclusion criteria:** (1) Major congenital malformation (2) Chromosomal aberration (3) Lack of informed parental consent (4) On enteral feeds for more than 12 hours (5) Contraindications for enteral feds (6) Life threatening illness/condition

**Outcomes:** The primary outcomes would be safety of the probiotic bacteria and their ability to colonise the gut (detected by serial stool cultures) and improve *Bifidobacterium breve* counts in stool. Secondary outcomes would include all cause deaths, ≥ Stage II NEC (Bell Staging)^12^, LOS (blood culture positive sepsis 72 hours after birth), and time to reach full enteral feeds (150ml/kg/day).

**Safety during clinical trial:** Sepsis from probiotic organisms is a possibility in preterm neonates given their immunocompromised status, and the frequent presence of indwelling catheters, foreign bodies and chronic illnesses. However despite isolated case reports (with favourable outcomes), no such untoward effects have been reported in any of the clinical trials of probiotic supplementation in premature babies. Safety will be assessed by monitoring (1) Blood culture positive sepsis by the probiotic bacteria and (2) Side effects such as abdominal distension, vomiting, and diarrhea leading to cessation of the supplementation. All outcomes as well as safety parameters will be monitored from enrolment in the trial till death or reaching the corrected age of 38 weeks.

Pre-planned subgroup: A subgroup analyses is planned for extremely preterm (Gestation < 27 weeks) neonates as they are at the highest risk for mortality and morbidities such as NEC, LOS, and feed intolerance compared to those with gestation ≥28 weeks.

**Randomisation, allocation concealment, and blinding:** Opaque, sealed, coded envelopes containing computer generated random numbers will be used for randomisation. Allocation concealment would be assured by formal enrolment of a neonate only after obtaining informed parental consent and registering the basic data. Randomisation will be stratified by neonatal gestational age (up to 27^+6^ weeks and **≥**28 weeks) to ensure that extreme preterm neonates are equally distributed between the two arms of the trial. Products of multiple pregnancies will be considered as separate individuals. The Clinical Trial Pharmacist (CTP) at KEM Hospital will supply the randomisation sequence and the sachets (identical design, weight, smell, and taste) containing either the probiotic (*Bifidobacteria breve* M-16V; 5 billion colony forming units per sachet with dextrin as carrier) or placebo (Equal volume of only Dextrin) manufactured by Morinaga Milk Industries, Japan, to the nursing staff. This assures masking of all investigators, nursing staff and parents with regards to the allocation status of enrolled neonates. The primary outcome (stool bacterial colony counts) will also be assessed in a blinded fashion by the laboratory in Sydney; to assure blinding and high quality of the trial.

**Probiotic** **protocol:** When ready for enteral feeds, enrolled neonates will be supplemented with the allocated sachet contents, and continued until the corrected age 37 weeks. The supplementation dose per day will be a reconstituted sachet volume containing 3 billion organisms per day given as a single dose irrespective of the gestation at birth. The dose and duration of supplement is based on the previous studies in preterm neonates.^5,10^ For preterm neonates who are on minimal enteral feeds (see attached protocol) the daily dose will be 1.5 billion organisms per day until reaching nutritional feeds of 50 ml/kg/day. Administration of the probiotic supplementation will be stopped during periods when the enteral feeds are stopped as per the discretion of the attending neonatologist. The common indications for stopping feeds include sepsis and NEC.

The manufacturer is not the sponsor but is only supplying the product free for the trial (Product information brochure attached).

**Duration of trial and enrolment:** Based on the current rates of admission at KEM hospital, this trial is expected to enrol 120 premature babies over a 6-8 month period.

Serial stool cultures: Two stool samples would be collected for quantitative cultures from each baby enrolled in the trial as follows: One before and one after 28 days of supplement administration. Based in Sydney, the investigator (PC) involved in stool culture studies will be masked to the allocation status of the enrolled neonate, assuring blinding of the primary outcome assessors.

Statistical approach: Group outcomes will be summarised using medians, interquartile ranges and ranges for continuous outcomes. Categorical outcomes will be summarised using frequency distributions. The tests used for comparisons would depend on the distribution of the data. The analysis will be based on the intention to treat principle.

Sample size estimation: Group sample sizes of 50 per group will achieve 90% power to detect the colonisation rate of 30% in probiotic group compared with the 5% rate in the control group when using a two-sided test of proportions with continuity correction at 5% significance level.^5^ An additional 20 neonates (10 in each arm) will be enrolled to cover for loss to follow up.

Regulatory considerations: Approval from the institutional ethics committee will be obtained for the trial protocol and the “Information for parents” for obtaining parental consent. The protocol will be registered under the Australian Clinical Trials Registry (ACTR). Clinical Trials Notification (CTN) approval will be obtained from Therapeutic Goods and Administration (TGA) authorities, Canberra.

**Data handling, storage, confidentiality:** The NHMRC Australia guidelines will be followed for confidentiality and data storage.

**Risk cover:** As suggested by the office of Prof John Newnham (Head: School of Women and Infant’s Health), the issue of risk cover will be handled by the office of Client Service Manager ([Graham.Metcalf@icwa.wa.gov.au](mailto:Graham.Metcalf@icwa.wa.gov.au), 08 9264 3371) after ethics approval has been obtained.

**References**

1. Millar M, Wilks M, Costeloe K. Probiotics for preterm neonates? Arch Dis Child Fetal Neonatal Ed 2003; 88: F 354-58.
2. Rautava S. Potential uses of probiotics in the neonate. Semin Fetal neonatal Med 2007;12 (1):45-53.
3. Martin CR, Walker WA. Probiotics: role in pathophysiology and prevention in necrotizing enterocolitis. Semin Perinatol. 2008; 32(2):127-37
4. Neu J, Douglas-Escobar M, Lopez M. Microbes and the developing Gastrointestinal tract. Nutr Clin Pract 2007; 22(2):174-82.
5. Deshpande G, Rao S, Patole S. Probiotics for prevention of necrotising enterocolitis in preterm neonates with very low birth weight- A systematic review of randomised controlled trials. Lancet 2007; 369(9573):1614-20.
6. Probiotics for prevention of necrotising enterocolitis in preterm infants. Alfaleh K, Bassler D. Cochrane Database Syst Rev 2008 ;(1):CD005496.
7. Barclay AR, Stenson B, Simpson JH et al. Probiotics for necrotizing enterocolitis: a systematic review. J Pediatr Gastroenterol Nutr. 2007;45(5):569-76
8. Deshpande G, Rao S, Patole S, Bulsara M. Updated meta analysis of probiotics for preventing necrotising enterocolitis in preterm neonates. Pediatrics 2010; 125(5): 921-30.
9. Satoh Y, Koichi S, Hikaru U et al. Bifidobacteria prevents necrotising enterocolitis and infection in preterm infants. International Journal of Probiotics and Prebiotics. 2007; 2 (2/3): 149-54.
10. Indrio F, Riezzo G, Raimondi F, et al. The effects of probiotics on feeding tolerance, bowel habits, and gastrointestinal motility in preterm newborns. J Pediatr. 2008;152(6):801-6.
11. Weisburg WG, Barns SM, Pelletier DA, Lane DJ (1991) 16S ribosomal DNA amplification for phylogenetic study. J Bacteriol. 173:697-703.
12. Walsh MC, Kliegman RM. Necrotising enterocolitis: treatment based on staging criteria. Pediatr Clin North Am 1986; 33: 179-201.

**Probiotic supplementation for reducing all cause mortality and definite necrotising enterocolitis in preterm very low birth weight neonates- Part 1: A randomised controlled pilot trial**

**Abbreviated title:** **PANTS** (Probiotics for Neonates) **trial**

***Information sheet for parent(s)***

Dear Parent(s)

We sincerely appreciate your efforts during this stressful time in reading the information about our research project. Take the time to read the information carefully before deciding whether you would like to take part in the research. Please feel free to ask any questions you may have about the study; contact details for the research team are given at the end of this sheet. The trial has been approved by the KEMH Ethics Committee, is registered with the Australian Clinical Trials Registry (ACTR) and the Therapeutic Goods Administration (TGA) office in Canberra. This study is sponsored by the “telethon 7” (Channel 7 Telethon trust).

Background to the study

Probiotics are the live beneficial microorganisms that are naturally present in the digestive tract. These bacteria promote health by suppressing the growth of potentially harmful bacteria, improving immune function, enhancing the protective barrier of the digestive tract, and helping us to produce vitamin K. Human beings have been consuming these bacteria for hundreds of years in the form of various food supplements, the commonest being Yogurt, and “Yakult” (containing the bacteria lactobacillus acidophilus). Immature bowel function in premature babies can cause feeding difficulties, diseases like necrotising enterocolitis (NEC- an inflammatory condition which attacks and kills the lining of the bowel), and infrequently, death. These conditions can delay the time it takes for the baby to tolerate full milk feeds and consequently increase the time spent in hospital.

Many clinical trials have evaluated the safety and benefits of probiotic supplementation in premature babies. Our team conducted an analysis of the results of those trials involving babies born at 33 weeks or earlier and less than 1.5 kg in body weight. The results indicated that the risk of death and NEC was reduced by 53% and 64% respectively in premature babies given probiotic supplement compared with control group babies. The time taken by premature babies to achieve full milk feeds was also less when given probiotic supplements, by an average of nearly three days. These results indicated the significant potential of probiotic supplementation in saving premature babies from a prolonged hospital stay, death and disease. Subsequent to this review, 4 more trials involving 783 premature babies have been published indicating the safety and efficacy of probiotic supplementation in this high-risk population. The results of our recently updated analysis reconfirm that probiotics reduce the risk of death and NEC by over 50% in premature babies while significantly reducing the time taken to establish full milk feeds. Experts have now commented that with a few precautions, the current evidence justifies routine use of probiotics in premature babies.

**If probiotics are so beneficial why are we doing this study and not offering them as a routine treatment?**

Based on the current evidence and the expert comments mentioned above, we believe that it is now difficult to deny the benefits of routine probiotics supplementation to premature babies. However non-availability of a well tested, tolerated, and safe product is the main hurdle towards providing probiotic therapy for premature babies in Australia. It is extremely important to confirm the identity of the probiotic bacteria and rule out potentially harmful contaminants and other ingredients in any commercial product before using it routinely in premature babies. It is equally important to confirm the ability of the specific probiotic bacteria in the product to colonise the gut of premature babies. Otherwise we will not know why the product did or did not work. Our proposed study is designed to address all these issues before offering probiotic supplementation as a routine for premature babies in our nursery.

Our research involves rigorous evaluation of the safety and efficacy of a probiotic product that is appropriate for premature babies. It will specifically evaluate whether the supplement is well tolerated by premature babies, and is effective in colonising their gut (as detected by stool specimen studies) without causing any significant adverse effects. If the product is shown to be well tolerated, safe and effective, the babies who are receiving the probiotic supplement stand to benefit in terms of reduction in the risk of death and disease like NEC, and a decrease in the time taken to reach full milk feeds.

**What will the study mean for you and your child?**

We are asking mothers of all babies born at KEMH before 33 weeks of pregnancy, and birth weight less than 1500grams to consider taking part in the study. We are conducting a “randomised, placebo-controlled trial” in premature babies to study the safety and efficacy (colonisation of gut detected in stool specimens) of probiotic supplementation in premature babies. Each baby will receive the probiotic or placebo (dextrin, a corn starch, in a minute amount) supplementation daily for a period until reaching 37 weeks corrected age. Two stool samples will be collected – prior to commencing and three weeks after commencement of the trial. We will collect information about your baby’s clinical progress from the information that is collected as part of normal care.

Can I change my mind during the study?

Participation in the study is voluntary and you may decide to withdraw your baby from the trial at any time. Should you decide not to take part, this will not interfere with your or your child’s medical care.

**Are there benefits in taking part in this research?**

Yes, there are potential benefits (reduced risk of death, NEC, and reduced time to full quota of milk feeds) if the randomisation process results in chance allocation of your baby to probiotic and not the placebo supplement. It is important to note that our study is designed primarily to evaluate whether the probiotic supplement is *safe and effective in colonising the gut* of premature babies. If that does turn out to be the case, it is possible that babies receiving the probiotic (not the placebo) supplement may benefit.

**Are there any disadvantages involved in taking part?**

Infection from “probiotic” bacteria is an uncommon but is a possible adverse effect that has been reported by some researchers. However it is important to note that none of the 11 clinical trials involving 2023 premature babies to date have reported any significant side effects of probiotic supplements including infection by probiotic organisms. Probiotic infections are easier to treat compared with those from highly toxic organisms.

We will be monitoring all babies enrolled in the trial very carefully for this possibility and treat them promptly with appropriate antibiotics if necessary. Other potential side effects of probiotic supplementation include abdominal distension, vomiting, and diarrhea. The independent safety monitoring committee will monitor for any significant adverse effects during the trial. Continuation in the trial will be reviewed immediately for any baby noted to have such side effects.

**Is there any pain or discomfort involved in the study?**

No, the administration of probiotic supplement *per se* is not expected to cause any pain or discomfort. There is no blood collection involved. As part of the study we will be collecting two stool specimens from all babies enrolled in the trial. All efforts are undertaken to minimise the pain and discomfort (from any cause) of your baby during his/her stay in the nursery.

How will information about my baby and family be stored?

We will give each baby a study number - all samples and information collected are identified by this number and not by your baby’s name. All information will be recorded confidentially and stored under current data protection guidelines. The participating researchers will abide by the terms of the *Code of Practice for the Use of Name-identified Data -NHMRC guidelines* and all data will be retained for a minimum of 5 years.

Please contact the study researchers A/Prof Patole or Prof. Simmer who will always be available (Tel: 9340 2003) to provide you with more information about the study if needed. If you have any concerns or complaints regarding this study, you can contact the Director of Medical Services at KEMH (Telephone No: (08) 9340 2222). Your concerns will be drawn to the attention of the Ethics Committee who is monitoring the study.

**Many thanks for taking the time to read this information leaflet. Please feel free to ask any questions.**

**If you would like to take part in the study, please sign the consent form provided.**
